# Effects of dietary palm olein on the cardiovascular risk factors in healthy young adults

**DOI:** 10.29219/fnr.v62.1353

**Published:** 2018-07-16

**Authors:** Chenyan Lv, Yifei Wang, Cui Zhou, Weiwei Ma, Yuexin Yang, Rong Xiao, Huanling Yu

**Affiliations:** 1 School of Public Health, Beijing Key Laboratory of Environmental Toxicology, Capital Medical University, Beijing, China; 2 National Institute for Nutrition and Health, Chinese Center for Disease Control and Prevention, Beijing, China

**Keywords:** palm olein, saturated fatty acids, dietary intervention, replacement, lipid metabolism

## Abstract

**Background:**

Dietary saturated fatty acids are always being hotly debated. Palm olein rich in saturated fatty acids (45.98%) is often considered as being atherogenic nutritionally. There is a lack of information on effects of dietary oil by partially replacing with palm olein on human health.

**Methods:**

A randomized controlled trial with 88 participants has been conducted to elucidate the effect of palm olein on cardiovascular risk factors.

**Results:**

By comparing the soybean oil group (saturated fatty acids amounted to 23.31%) with the cocoa butter group (saturated fatty acids amounted to 93.76%), no significant difference was found (*p* > 0.05) in physiological parameters, serum oxidative stress levels, inflammatory factor, glucose metabolism, and lipid profiles of subjects, which are all cardiovascular risk factors. Although results showed that intervention time can influence the cardiovascular risk factors significantly (*p* < 0.05), there is no relationship between intervention time and dietary oil type.

**Conclusion:**

Therefore, partial replacement of dietary oil by palm olein may not affect cardiovascular risk factors in healthy young adults. There are differences between our research and previous researches, which may be due to the different amount of palm olien in diet. Our research will provide a solid foundation for the application of palm olein in human diets and in the food industry.

Fat and oil are important components in nutrition for animals and human beings. Dietary fats and oils perform a variety of functions. They provide energy and certain nutrients such as lipid-soluble vitamin, and maintains your core body temperature (1). Recently, several researches have indicated that poly-unsaturated fatty acids (PUFA) have affected many aspects of immune system, including antibody production by B cell and better health of gut-associated lymphoid tissue (2). PUFA has been proved to reduce the production of low-density lipoprotein cholesterol (LDL-C) and increase the production of high-density lipoprotein cholesterol (HDL-C) (3). Meanwhile, both the lipid content and the type of fatty acids can cause alterations both in liver and white adipose tissues. In contrast to unsaturated fatty acids, dietary saturated fatty acids (SFA) have been proved to affect the body health. For example, once the 18:2 fatty acids were replaced by 16:0, the total cholesterol level and LDL–HDL ratio increased slightly, indicating the adverse effect of saturated fatty acids (4). Several animal experiments and controlled feeding studies in humans have demonstrated increases in fasting levels of TC and LDL-C after SFA consumption (5). Moreover, the North Karelia study has demonstrated that reduction of SFA in diet can decrease the serum cholesterol and result in substantial reduction of cardiovascular disease (CVD) risk (6). However, recent systematic reviews on prospective cohort studies have questioned the relationship between dietary SFA and CVD. The observational studies indicated that overall SFA intake does not increase the risk for stroke (7). Another cohort study on Dutch population has indicated that higher SFA intake was not associated with higher ischemic heart disease (8). Therefore, the relationship between dietary SFA and cardiovascular risk factors needs to be further identified. Both the type and the amount of dietary SFA may be key factors for CVD.

Palm olein, a vegetable oil obtained from the palm tree fruits, is composed of ~50% palmitic acid, 40% oleic acid, and 10% linoleic acid. Palm olein with a high content of the SFA (palmitic acid) and a low content of unsaturated fatty acid has been proved to increase the serum cholesterol concentrations in humans since 1960s (9). It has been demonstrated that there is no clear health benefit of replacing saturated fatty acids with starchy food, whereas replacement with unsaturated fatty acids reduced the risk of CVD (10). A decrease as slight as 5% in dietary saturated fatty acid amounted to 13% decrease in CVD occurrence and 26% decrease in deaths (11). On replacing the ‘red oil’ (palm oil) with the ‘white oil’ (branded vegetable oil), the saturation of dietary oil will be reduced by about 30% (12). However, a randomized 30-d/30-d crossover study comparing the effect of palm olein and olive oil on plasma lipids has indicated that palm olein is not always a plasma cholesterol-raising fatty acid (13). Similarly, a recent meta-analysis has indicated that the favorable and unfavorable changes in coronary heart disease (CHD)/CVD risk markers are both observed when palm oil constitutes the main dietary SFA intake (14). Recently, a randomized trial of the effects of hybrid palm oil supplementation on human plasma lipid patterns has provided additional support for the concept that hybrid palm oil can be seen as the ‘tropical equivalent of olive oil’ (15). These studies indicated that the health effect of palm oil is still controversial. Whether partially replacing the dietary oil such as soybean oil with palm olein will affect the body health or not remains unknown. The association between palm olein intakes with CVDs needs further identification, especially for the healthy young adults.

In this study, a single-blind, randomized parallel feeding intervention trial has been employed to determine the influence partially replacing dietary oil with palm olein on the cardiovascular risk factors and further investigate whether the dietary oil composition and the high ratio of saturated fatty acids can affect healthy young adults.

## Materials and methods

### Materials

Palm olein used in this study was purchased from Tianjin julong jiahua investment group co., Ltd., China. Cocoa butter was purchased from Zhejiang Qili xingguang cocoa products Corp., Ltd., China. Soybean oil was purchased from Shanghai Liangyou Haishi oils & fats industry co., Ltd., China. All the dietary oils met the national standards (Soybean oil, GB1535-2003; palm olein, GB15680-2009; Cocoa butter, GB/T 22000-2006) and production licenses were obtained. Vacuum blood needle and related materials used in this study were purchased from Becton, Dickinson and Company, USA.

### Justification of sample size

A sample size of 108 participants was proposed by using PASS 11.0.7; we conducted a post hoc power calculation and estimated that a sample size of 108 participants would enable the detection of a minimum between palm olein and high oleic sunflower (HOS), in cholesterol of 0.2% (80% power, α = 0.05) (16). Our sample size was reasonable according to similar studies of palm olein intervention (17–19).

### Subjects

A convenience sample of 108 students from Capital Medical University (50 men and 58 women; age 21.59 ± 0.39; body mass index [BMI] 21.03 ± 0.37; mean ± S.E) was selected to participate in the study. The volunteers with underlying diseases have been ruled out (*n* = 15).

The subjects were randomly allocated to three groups and they were treated with different diets. Data regarding 88 of the volunteers who completed the entire study are included in this report. All of the volunteers were recruited from Capital Medical University by the recruiting announcement in February, 2016. The criteria for inclusion and exclusion are listed below in detail. (1) Inclusion criteria: Adult males or females aged 20–40 years; BMI (18.5–23.9); subjects without a history of atherosclerotic disease or hypertension; subjects must understand the study and then agree to participate; subjects must adhere closely to prescribed food consumption as per research protocol. (2) Exclusion criteria: Abnormal liver function test (elevated transaminases – alanine transaminase [ALT], aspartate transaminase [AST])/abnormal kidney function test (elevated plasma creatinine [CR]); history of type 2 diabetes mellitus, cancer, stomach ulcers, drug abuse or alcoholism; smokers; on lipid/blood pressure-lowering medication/supplements; blood pressure > 140/90 mm Hg; fasting cholesterol (CHO) > 6.2 mmol/L; fasting triglyceride (TG) > 2.0 mmol/L; candidates who are going abroad during the planned schedule for the dietary intervention; subject must not be allergic to intervention; pregnancy and breastfeeding; subjects taking nutritional supplements or on any weight-loss programs; female subjects who are on oral contraceptives; subjects with a history of hypo- and hyperthyroidism; subjects with constipation/bowel movement problems. Finally, 93 subjects were randomized and divided into three groups according to BMI using simple randomization adjusted with sex and ages.

This study was conducted according to the Declaration of Helsinki and was approved by the Ethics Committee for Clinical Research in Chinese Nutrition Society (Approval No. CNS2016001). All of the subjects signed the informed consent document once they learned of the purpose, procedures, and risks of this study. This study has been registered on the Chinese Clinical Trial Registry and the registry number is ChiCTR-IIR-16008733.

### Experimental design

**Fig. 1:**
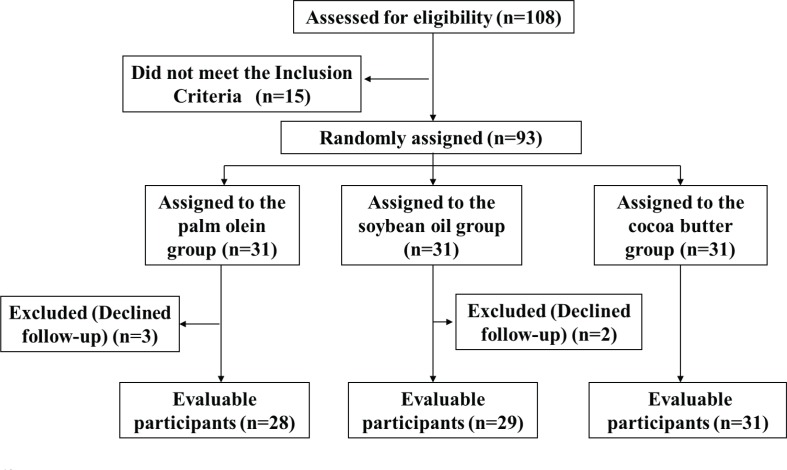
Study flow diagram.

This study was a single-blind, randomized parallel feeding intervention trial. The subjects were randomly assigned to three groups (cocoa butter group, palm olein group, and soybean oil group) (Fig. 1). This study was conducted for 16 weeks (from April, 2016, to July, 2016). Subjects were then randomized to one of the three treatments for a period of 16 weeks in parallel. The study consisted of an initial stabilization period of 1 week and intervention period of 16 weeks. During the stabilization period, all subjects consumed the basal diet. The basal diet consisted of vegetables, meat, and rice, and only soybean oil was used to prepare the basal diet. As for the intervention period, subjects from three groups were intervened with test fats.

All food (vegetables and staple food) in the diet was cooked with soybean oil. And the oil used for staple food (walnut cake, ujube cake, or Cantonese sponge cake) was partially replaced with cocoa butter and palm olein. The compositions of staple food are shown in Table S1. Due to the different amounts of oil used for making walnut cake, ujube cake, and Cantonese sponge cake, test meals were served as breakfast (for walnut cake) or breakfast (for ujube cake) and lunch (for Cantonese sponge cake). The macronutrient compositions of the test diets were identical (e.g. % of energy as carbohydrates, protein, and fat). Based on the dietary survey of the subjects before intervention, intake amounts of fatty acid from the diet of subjects are 30–36 g/day. In this study, we have replaced 20–22 g oil in the diet with test fatty acids, which is about 1/5 of the total dietary fat. The fatty acid compositional analysis is shown in Table 1. The ratio of saturated fatty acids of cocoa butter, palm olein, and soybean oil is about 4:2:1. And subjects were provided with uniform calorie meals prepared with fats 5 working days a week. At the weekends, meals were not provided to the subjects, instead dietary advice was given including the restricted intake of snacks. All the test meals were provided by the canteen and all the subjects took the meals in the canteen, which were supervised by us. After the meal, we weighed the remaining amount of test meals of subjects to guarantee the intake of calories was equal.

**Table 1:** The compositional analysis of palm olein, soybean oil, and cocoa butter

Fatty-acid composition^a^		Palm olein (%)	Soybean oil (%)	Cocoa butter (%)
Saturated fatty acid	sn-1,3	45.98	23.31	93.76
	12:0	0.3	0	0
	14:0	1.1	0	0.08
	16:0	29.4	11.1	26.2
	18:0	3.4	3.8	37.1
	20:0	0.3	0.4	1.1
	22:0	0	0.6	0
Mono-unsaturated fatty acid	sn-1,3	44.43	27.73	5.8
	16:1	0.4	1.5	0
	18:1	50.3	22.4	36.0
	20:1	0	0.1	0
	22:1	0	0.7	0
Poly-unsaturated fatty acid	sn-1,3	9.59	48.96	0.44
	18:2	14.5	51.7	2.0
	18:3	0.3	6.7	0

a Concentration of fatty acids were performed by % by wt.

### Diets

The mean daily calorigenic nutrient intakes from the cocoa butter, palm olein, and soybean oil diets during the intervention periods are shown in Table 2. Nutrient intake from test fat was similar for both the stabilization and intervention periods. The test meals provided about 35% energy as fat, 49% as carbohydrates, and 16% as proteins.

**Table 2:** Daily energy intake from three calorigenic nutrients during dietary intervention

Energy/calorigenic nutrients	Palm olein	Soybean oil	Cocoa butter
Total energy (kcal)	1807.58 ± 316.43	1749.28 ± 445.37	1786.09 ± 323.83
Protein (% of energy)	15.57 ± 1.69	15.53 ± 2.45	16.03 ± 2.68
Lipid (% of energy)	35.56 ± 3.86	35.42 ± 4.96	35.90 ± 4.43
Carbohydrate (% of energy)	48.87 ± 4.33	49.04 ± 5.73	48.07 ± 5.51

### Blood sampling

Before the study was started, every subject received a random inclusion number that was used for identification and labeling of blood and serum tubes. Blood samples were drawn after an overnight fast immediately before breakfast at the end of each period. Baseline samples were collected during the stabilization period. Serum was separated by centrifugation after sampling to avoid hemolysis and then stored at −80°C until analyzed. Sample treatments were blinded to the analysts.

### Laboratory methods

The energy of test diets was calculated according to the China Food Composition. Blood from overnight fasting subjects was collected by antecubital venipuncture into vacutainer tubes containing EDTA for lipid profile determinations and no anticoagulant for apolipoprotein determinations once 4 weeks. The fasting serum CHO, high-density lipoprotein cholesterol (HDL-C), low-density lipoprotein cholesterol (LDL-C), and triglyceride (TG) were measured using commercial kits (Ying-Ke-Xin-Chuang Science and Technology Ltd, Xiamen, China) on an automatic biochemical analyzer. ApoA, ApoB, ApoE, Lp(a), and hsCRP were determined by immunoassay. Nonesterified fatty acids (NEFA) were determined by colorimetry. CR, ALT, and AST were tested by enzymatic methods. The oxidative stress parameters (malondialdehyde [MDA], glutathione peroxidase [GSH-px], total antioxigenic capacity [T-AOC], glutathione [GSH], and oxidized glutathione [GSSG]) were determined by the test kits from Nanjing Jiancheng Bioengineering Institute. Plasma concentrations of insulin and leptin were measured using ELISA kit (R&D Company, Germany and Phoenix pharmaceuticals Inc., USA).

### Data analysis

All the data were analyzed by SPSS 22.0. Due to the study design, we have tested the index with various time points. Herein, the data were analyzed by analysis of variance for repeated data. All the data were shown as mean ± SE. Probability values less than 0.05 were considered to indicate a significant difference.

## Results

### The effects of dietary palm olein on physiological parameters of the subjects

The BMI of the subjects (41 men, 47 women) in the three groups ranged from 20 to 22, which is within the normal range of the Chinese standard (18.5–23.9). The BMI in palm olein group has not changed significantly during the 16-weeks intervention, similar to the cocoa butter group and the soybean oil group. There is no significant difference (*p* > 0.05) among three groups as well (Table 3). Similar to BMI, no obvious change has been observed for the body fat rate (BFR) in palm olein group during intervention (Table 3), which is the same for soybean oil group and cocoa butter group.

**Table 3:** The changes of BMI, BFR, CR, ALT, and AST level during intervention and the effect of palm olein on them

Index	Time (week)	Group^a^			*P*_(Time)_	*P*_(Group)_
		Cocoa butter (*n* = 31)	Palm olein (*n* = 28)	Soybean oil (*n* = 29)		
BMI (kg/cm^2^)	0	21.12 ± 0.30	21.50 ± 0.46	20.48 ± 0.35	0.000	0.246
	8	21.22 ± 0.28	21.52 ± 0.44	20.71 ± 0.30		
	16	21.03 ± 0.29	21.06 ± 0.45	20.44 ± 0.31		
BFR (%)	0	22.72 ± 1.28	23.02 ± 1.56	21.60 ± 1.36	0.000	0.775
	8	21.55 ± 1.35	22.09 ± 1.50	20.56 ± 1.29		
	16	21.91 ± 1.32	21.69 ± 1.41	20.76 ± 1.31		
CR (μM)	0	60.81 ± 2.07	61.68 ± 2.37	61.10 ± 2.17	0.000	0.742
	8	60.26 ± 1.96	62.32 ± 2.47	60.38 ± 2.07		
	16	66.29 ± 2.37	69.5 ± 2.89	65.76 ± 2.17		
ALT (U/L)	0	11.77 ± 1.15	13.39 ± 1.69	10.93 ± 0.78	0.175	0.527
	8	11.48 ± 1.16	11.75 ± 1.57	9.79 ± 0.66		
	16	11.71 ± 1.16	12.14 ± 1.64	11.17 ± 0.99		
AST (U/L)	0	14.48 ± 1.33	15.93 ± 1.20	21.60 ± 1.36	0.000	0.434
	8	20.03 ± 0.99	22.04 ± 2.51	20.56 ± 1.20		
	16	13.81 ± 1.38	14.14 ± 1.22	20.76 ± 1.06		

a The data were shown as mean ± SE.

The results for CR level are shown in Table 3. The serum CR level in palm olein group increased slightly (from 60 μmol/L to 66 μmol/L) after 8 weeks’ intervention. There is a significant difference among the CR levels at different time points (*p* < 0.05). There is no interaction between the time and grouping factors. There is no significant difference (*p* > 0.05) among three groups (Table 3) and the grouping is not the influencing factor for CR levels. The levels of liver function indicators ALT and AST are shown in Table 3. The ALT level has not changed significantly along with time. There is no interaction between time and grouping factors. However, the AST level increased significantly after 8 weeks’ intervention, and then decreased after 16 weeks’ intervention (Table 3), which means time is an influencing factor for AST level. There is no significant difference (*p* > 0.05) among three groups (Table 3) due to ALT and AST.

### The effects of dietary palm olien on the levels of serum oxidative stress and inflammatory factors

In order to elucidate whether dietary palm olein can affect the oxidative stress response, the levels of GSH-px, GSH, GSSG, MDA, and T-AOC have been identified. There is a significant difference among the GSH-px levels at different time points (*p* < 0.05) (Table 4). There is no interaction between the time and grouping factors (*p* = 0.19 > 0.05). The result of soybean oil group is the same as palm olein group. Grouping has not affected the GSH-px level significantly (*p* = 0.28 > 0.05).

**Table 4:** The effects of palm olein on the oxidative stress levels (GSH-PX; GSH; GSSG; GSH/GSSG; MDA; T-AOC; hsCRP) during dietary intervention

Index	Time (week)	Group^a^			*P*_(Time)_	*P*_(Group)_
		Cocoa butter (*n* = 31)	Palm olein (*n* = 28)	Soybean oil (*n* = 29)		
GSH-px (U/ml)	0	277.01 ± 25.13	218.95 ± 26.93	218.55 ± 14.97	0.001	0.283
	8	253.02 ± 15.20	236.70 ± 14.97	265.75 ± 17.73		
	16	194.59 ± 14.53	202.62 ± 16.44	216.79 ± 13.86		
GSH (μM)	0	3.25 ± 0.31	3.53 ± 0.25	2.71 ± 0.20	0.707	0.787
	8	3.17 ± 0.25	2.81 ± 0.22	3.15 ± 0.24		
	16	3.12 ± 0.24	3.01 ± 0.22	2.91 ± 0.24		
GSSG (μM)	0	2.47 ± 0.11	2.16 ± 0.10	2.46 ± 0.09	0.000	0.884
	8	2.31 ± 0.12	2.58 ± 0.10	2.41 ± 0.12		
	16	3.06 ± 0.12	3.16 ± 0.12	3.11 ± 0.10		
GSH/GSSG	0	1.57 ± 0.229	1.90 ± 0.21	1.23 ± 0.14	0.157	0.268
	8	1.65 ± 0.20	1.22 ± 0.14	1.53 ± 0.18		
	16	1.40 ± 0.40	1.23 ± 0.31	1.03 ± 0.12		
MDA (μM)	0	5.16 ± 0.23	6.52 ± 0.43	5.00 ± 0.28	0.237	0.092
	8	5.33 ± 0.26	4.97 ± 0.20	5.27 ± 0.30		
	16	5.03 ± 0.31	5.25 ± 0.46	5.09 ± 0.35		
T-AOC (U/ml)	0	12.77 ± 0.42	12.82 ± 0.58	12.18 ± 0.61	0.000	0.261
	8	15.18 ± 0.40	15.58 ± 0.53	15.14 ± 0.51		
	16	13.36 ± 0.51	14.73 ± 0.72	12.77 ± 0.38		
hsCRP (mg/L)	0	3.52 ± 0.70	4.66 ± 0.71	3.32 ± 1.05	0.000	0.296
	8	0.36 ± 0.06	1.09 ± 0.41	0.48 ± 0.06		
	16	1.81 ± 0.33	1.36 ± 0.26	1.36 ± 0.19		

a The data were shown as mean ± SE.

There is no significant difference (*p* = 0.46 > 0.05) between the GSH levels in palm olein group and soybean oil group (Table 4), and the GSH levels didn’t change significantly along with time (*p* = 0.71 > 0.05). On the contrary, the GSSG levels increased significantly in palm olein group along with intervention time (*p* < 0.05) (Table 4). There is a significant interaction between time and grouping factors. Grouping is not an influencing factor for GSSG levels either (*p* > 0.05). In order to better know the antioxidant ability, we have calculated the GSH/GSSG. As shown in Table 4, the GSH/GSSG level in palm olein group decreased significantly after 8 weeks’ intervention. Whereas, the GSH/GSSG level in cocoa butter group and soybean oil group increased slightly after the 8 weeks’ intervention and decreased after the 16 weeks’ intervention (Table 4).

MDA as a metabolite of PUFA has been identified and the results are shown in Table 4. After 16 weeks’ intervention, all of three groups didn’t change significantly along with intervention time. However, there is an interaction between intervention time (*p* < 0.05) and the type of dietary fatty acids and there is no significant difference in MDA among three groups (*p* > 0.05). In addition, the T-AOC level changed significantly along with time (*p* < 0.05), which increased during the first 8 weeks’ intervention and decreased slightly after another 8 weeks’ intervention. There is no interaction between intervention time and the types of dietary oil (*p* > 0.05) and there is no significant difference in T-AOC level among these three groups (*p* > 0.05) (Table 4).

hsCRP as an inflammation marker has been identified to know the effect of palm olein on body’s inflammatory response. As shown in Table 4, the hsCRP level in the palm olein group decreased significantly after 8 weeks’ intervention and then increased slightly. Similar to almost all of the results, there is no significant difference among these three groups (*p* > 0.05), indicating that the type of dietary oil may not affect the hsCRP level.

### The effects of dietary palm olein on the glucose metabolism

To evaluate the effect of palm olein intake on the glucose metabolism, the levels of fasting blood-glucose, insulin, and leptin have been determined and the results are shown in Table 5. The fasting blood-glucose level in palm olein group changed significantly during the intervention period (*p* < 0.05). There is no interaction between the intervention time and the type of dietary oil (*p* > 0.05). No significant difference was observed among these three groups (Table 5). Similarly, the insulin levels in the three groups decreased significantly along with time (*p* < 0.05) and there is no significant difference among three groups (*p* > 0.05) (Table 5). The results for homeostasis model assessment of insulin resistance (HOMA-IR) were shown in Table 5. The HOMA-IR ranged from 0.9 to 1.7 which is within the normal range and the trends for HOMA-IR are the same as the trends for insulin. Similarly, as shown in Table 5, the leptin level changed significantly along with time (*p* < 0.05). No significant difference has been observed among different groups (*p* > 0.05).

**Table 5:** The effects of palm olein on the glucose/energy metabolism (fasting blood-glucose; insulin; HOMA-IR; leptin) during dietary intervention

Index	Time (week)	Group^a^			*P*_(Time)_	*P*_(Group)_
		Cocoa butter (*n* = 31)	Palm olein (*n* = 28)	Soybean oil (*n* = 29)		
GLU (mM)	0	4.51 ± 0.05	4.67 ± 0.09	4.60 ± 0.06	0.000	0.514
	8	4.84 ± 0.07	4.89 ± 0.08	4.86 ± 0.06		
	16	4.30 ± 0.13	4.36 ± 0.13	4.24 ± 0.12		
INS (μIU/ml)	0	8.16 ± 0.52	7.62 ± 0.48	7.69 ± 0.64	0.000	0.883
	8	5.67 ± 0.45	4.85 ± 0.42	5.03 ± 0.44		
	16	6.41 ± 0.62	6.10 ± 0.49	6.78 ± 0.76		
HOMA-IR	0	1.64 ± 0.11	1.58 ± 0.11	1.60 ± 0.15	0.000	0.748
	8	1.22 ± 0.10	1.05 ± 0.10	1.08 ± 0.10		
	16	1.26 ± 0.14	1.21 ± 0.11	1.27 ± 0.13		
Leptin (ng/ml)	0	9.66 ± 1.45	8.98 ± 1.31	9.59 ± 1.77	0.004	0.742
	8	7.71 ± 1.22	7.12 ± 1.13	7.50 ± 1.25		
	16	11.04 ± 1.42	8.31 ± 1.10	8.74 ± 1.74		

a The data were shown as mean ± SE.

### The effects of dietary palm olein on the serum lipid profiles

Dietary fatty-acid composition and type can regulate lipids and lipoprotein metabolism. To know whether the dietary palm olein (rich in saturated fatty acids) can affect the blood lipid metabolism, the serum CHO, TG, LDL-C, and HDL-C have been determined every 4 weeks. As shown in Table 6, the intervention time may affect the CHO levels significantly (*p* < 0.05). However, no interaction has been observed between the intervention time and the dietary oil type. There are no significant differences in CHO level among cocoa butter, palm olein, and soybean oil groups either (*p* > 0.05). In contrast, the TG level did not change significantly along with time (*p* > 0.05) and the palm olein in the diet may not affect the TG level significantly (*p* > 0.05) (Table 6). Similarly, by the ANOVA of repeated data, the serum HDL-C and LDL-C level have changed significantly during 16 weeks’ intervention (*p* < 0.05) (Table 6). A significant difference (*p <* 0.05) has been observed for LDL-C levels among three groups. However, the difference may be due to the grouping factor at the beginning of the study. The trends during intervention are almost the same for three groups and there is no significant difference in HDL-C among them (*p* > 0.05). No interaction has been observed between the intervention time and different dietary oils in diets.

**Table 6:** The effects of palm olein on the serum lipid profiles (CHO; TG; HDL-C; LDL-C; Apo A1; ApoB; ApoE; NEFA; Lp(a)) during dietary intervention

Index	Time (week)	Group^a^			*P*_(time)_	*P*_(group)_
		Cocoa butter (*n* = 31)	Palm olein (*n* = 28)	Soybean oil (*n* = 29)		
CHO (mM)	0	4.74 ± 0.12	4.55 ± 0.80	4.39 ± 0.12	0.000	0.104
	4	4.78 ± 0.12	4.44 ± 0.74	4.56 ± 0.14		
	8	4.63 ± 0.12	4.27 ± 0.68	4.40 ± 0.13		
	12	4.65 ± 0.12	4.22 ± 0.49	4.59 ± 0.14		
	16	4.29 ± 0.12	4.30 ± 0.56	3.96 ± 0.11		
TG (mM)	0	0.91 ± 0.07	0.88 ± 0.08	0.77 ± 0.05	0.009	0.08
	4	0.94 ± 0.07	1.13 ± 0.17	0.88 ± 0.05		
	8	0.90 ± 0.05	0.98 ± 0.08	0.73 ± 0.04		
	12	0.93 ± 0.07	0.89 ± 0.08	0.76 ± 0.04		
	16	0.96 ± 0.09	0.87 ± 0.08	0.73 ± 0.04		
HDL-C (mM)	0	1.69 ± 0.06	1.65 ± 0.06	1.69 ± 0.06	0.000	0.672
	4	1.66 ± 0.06	1.60 ± 0.06	1.69 ± 0.06		
	8	1.80 ± 0.06	1.69 ± 0.06	1.73 ± 0.06		
	12	1.42 ± 0.04	1.39 ± 0.04	1.40 ± 0.05		
	16	1.46 ± 0.04	1.44 ± 0.05	1.45 ± 0.06		
LDL-C (mM)	0	2.49 ± 0.09	2.36 ± 0.13	2.24 ± 0.09	0.000	0.031
	4	2.38 ± 0.10	2.35 ± 0.10	2.23 ± 0.08		
	8	2.47 ± 0.10	2.31 ± 0.09	2.15 ± 0.07		
	12	1.95 ± 0.08	1.87 ± 0.09	1.72 ± 0.05		
	16	2.21 ± 0.10	2.24 ± 0.11	2.00 ± 0.08		
ApoA1 (g/L)	0	1.44 ± 0.06	1.39 ± 0.05	1.38 ± 0.05	0.010	0.354
	8	1.48 ± 0.06	1.40 ± 0.05	1.37 ± 0.05		
	16	1.38 ± 0.04	1.37 ± 0.04	1.29 ± 0.05		
ApoB (g/L)	0	0.75 ± 0.02	0.71 ± 0.03	0.68 ± 0.03	0.000	0.309
	8	0.70 ± 0.02	0.64 ± 0.03	0.61 ± 0.02		
	16	0.62 ± 0.03	0.68 ± 0.05	0.62 ± 0.04		
ApoE (mg/dL)	0	4.51 ± 0.20	4.51 ± 0.33	4.39 ± 0.22	0.000	0.515
	8	4.90 ± 0.21	4.57 ± 0.28	4.41 ± 0.23		
	16	4.11 ± 0.20	4.05 ± 0.27	3.66 ± 0.21		
NEFA (μM)	0	329.19 ± 28.59	297.61 ± 31.51	306.07 ± 32.09	0.000	0.397
	4	339.55 ± 42.98	290.11 ± 27.97	304.14 ± 24.01		
	8	391.35 ± 21.77	387.71 ± 20.93	435.58 ± 23.19		
	12	271.00 ± 32.00	238.61 ± 22.46	351.93 ± 40.46		
	16	345.03 ± 27.48	361.46 ± 38.49	360.55 ± 37.01		
LP(a) (mg/L)	0	219.42 ± 40.77	202.96 ± 41.89	181.38 ± 37.82	0.021	0.811
	4	190.94 ± 34.09	217.61 ± 44.67	171.17 ± 38.77		
	8	197.16 ± 37.30	189.00 ± 38.78	187.39 ± 44.78		
	12	225.32 ± 41.26	191.50 ± 39.99	159.03 ± 34.43		
	16	161.84 ± 28.17	167.61 ± 37.17	149.10 ± 31.54		

a The data were shown as mean ± SE.

The serum ApoA1, ApoB, and ApoE levels are shown in Table 6. Through ANOVA of repeated data, the intervention time has affected the levels of ApoA1, ApoB, and ApoE significantly (*p* < 0.05). Similar to the results for CHO, dietary palm olein intervention has not affected the ApoA1 level. The serum ApoA1 and ApoB levels decreased along with time after intervention (Table 6). In addition, the ApoE level in palm olein group was almost the same during 8 weeks’ intervention and then decreased slightly after that (Table 6). As for normal population aged 20–40, we have not observed significant changes in ApoA1, ApoB, and ApoE levels among cocoa butter group, palm olein, and soybean oil groups (*p* > 0.05).

As shown in Table 6, the NEFA level increased slightly during 8 weeks’ intervention and then decreased until 12 weeks, indicating that the intervention time can affect the NEFA level significantly (*p* < 0.05). There is no obvious difference in NEFA level among three groups (*p* > 0.05), which is similar to results for TG, CHO, LDL-C, and HDL-C. Meanwhile, after 4 weeks’ intervention, Lp(a) level in palm olein group is higher than the level of soybean oil group and cocoa butter group. Nevertheless, the Lp(a) level of palm olein group is between the level of cocoa butter group and soybean oil group after 12 weeks’ intervention. Thus, there is no significant difference (*p* > 0.05) among these three groups.

## Discussion

Dietary fatty acids have been regarded as the key risk factors for several chronic diseases, such as obesity, coronary heart diseases, and atherosclerosis. Several studies have shown that the composition of dietary oil may affect the blood lipid profiles (20), but the directions of the effects were inconsistent. In order to elucidate the effect of dietary oil with high ratio of saturated fatty acids on the cardiovascular risk factors, we have identified the physiological parameters, oxidative stress, inflammatory factors, glucose metabolism, and the serum lipid profiles during palm olein intervention.

Firstly, we have not observed the change of BMI and BFR among three groups, results of which indicate that the dietary oil partially replaced with palm olein may not affect the body weight and fat deposition. Analogously, there is no effect on the level of CR, AST, and ALT, suggesting that the dietary oils have not affected the function of kidney and liver. However, these results are contrary to the animal study of atherogenic diet based on saturated fat, which has shown a weight gain effect in rats and elevated AST and ALT level of rats (21), which can be attributed to the different amounts of saturated fat.

Previous research has suggested that oxidative stress plays a significant role in the decrease in beta-cell secretory function by exposure to free fatty acids for a long time (22). Meanwhile, the palm olein has been proved to exhibit protective effects on cardiovascular systems by attenuating the free radicals and then decreasing the oxidative stress (23). However, the oxidative stress indices exhibited no change during dietary intervention, indicating that palm olein and cocoa butter high in saturated fatty acids have no effect on the oxidative stress status for human body. This difference may be from different study subjects and intervention times. Interestingly, palm olein rich in tocotrienol fractions has been proved to reduce the levels of oxidative stress markers, which can be attributed to the effect of tocotrienol (24). Therefore, dietary palm olein may not have an adverse effect on oxidative stress relating to CVD. In addition, C-reactive protein (CRP) is associated with CVD and inflammation in apparently healthy individuals. Our study suggested that palm olein in diet has no significant effect on the hsCRP level (*p* > 0.05). This result is similar to a recent research on cocoa butter, which stated that dietary cocoa butter does not alter the inflammatory markers such as postprandial hsCRP in healthy women (25). Thus, dietary oil partially replaced with palm olein rich in saturated fatty acids has not affected the inflammation status of human body. In addition, the hsCRP levels after 8 weeks and 16 weeks of intervention are lower than the level before intervention, which may be due to the diet low in salt and oil.

What’s more, the fasting blood-glucose level has not been affected by palm olein, indicating that partially replacing dietary oil with palm olein may have little effect on the glucose metabolism. In addition, the insulin level and HOMA-IR decreased along with time, which may be due to the healthy dietary pattern (a regular diet low in salt, and with energy balance) during intervention. This result is in accordance with previous researches that palm based oil diets did not affect the markers of insulin resistance and glucose tolerance in overweight Malaysian adults (26). Thus, we can speculate that the moderate level of saturated fatty acids in diet has no relationship with insulin resistance. Similarly, palm olein replacement has not influenced the leptin level, indicating the palm olein intervention may not affect the energy metabolism.

Generally, no significant difference was observed for the blood lipid profiles during intervention for cocoa butter, palm olein, and soybean oil groups. In addition, CHO, TG, HDL-C, and LDL-C have not changed obviously during 16 weeks’ intervention. Previous researches on different vegetable oils have indicated that the CHO level in all treated rats for 8 weeks were within the normal range and there was no significant difference in CHO, which is similar to our results (27). However, the TG levels in palm olein group were significantly increased compared to control group (27), which is contradictory to our results. This can be explained by different objects and intervention time. At the same time, HDL-C and LDL-C as possible risk factors for coronary heart disease have not changed as well. This result is consistent with the results from positional distribution of fatty acids on dietary TGs, which showed that the ratio of HDL-C to LDL-C cholesterol and serum TG concentration of human body were unchanged after 3 weeks’ intervention (28). On the contrary, previous studies on the fats with a higher proportion of palmitic acid in the sn-2 position have indicated to decrease postprandial lipemia in healthy subjects (29). This can be attributed to the difference in intervention time.

As we have not observed the change in lipid profiles, whether the intervention can affect the apolipoprotein needs to be further identified. ApoA1 has been proved to negatively relate with HDL-C, and ApoB was negatively correlated with LDL-C (30, 31). Thus, results for ApoA1 and ApoB are similar to the results of HDL-C and LDL-C. Dietary intervention with cocoa butter, palm olein, and soybean oil has not affected the serum ApoE level. It has been proved that APOE3 is associated with the potential to more efficiently harvest dietary energy and to deposit fat in adipose tissue, while APOE4 carriers tend to increase fatty-acid mobilization and utilization as fuel substrates especially under high-fat intake (32). As a consequence, the dietary intervention has no effect on fat deposition and fatty-acid utilization.

The dietary intervention with palm olein has not affected the serum NEFA levels, possibly indicating that the high ratio of saturated fatty acid has not affected the adipose fat deposition. Similarly, previous results have shown that alterations in the dietary lipid intake of rats affected the composition but not the amount of myocardial NEFAs (33). Whereas pervious study has reported that Omega-3 supplementation caused a significant reduction in NEFA in the intervention group compared with the placebo group (34). Therefore, the composition of dietary fatty acids is vital for the levels of NEFA.

Meanwhile, Lp(a) as a low-density lipoprotein particle with a specific protein attached to it is hardly influenced by diet (e.g. low-caloric, low-fat, or high-cholesterol diets), which is in accordance with our results. However, previous reports on hydrogenated fish oil, partially hydrogenated soybean oil and butter have indicated the unfavorably effect on lipid risk indicators for coronary heart disease, especially the serum lipoproteins and Lp(a) in men (*p* < 0.02) (35). This may be due to the various intervention time and methods.

In this study, we have not observed the significant difference among three groups. The possible reasons for this observation may be that the sample size of 108 may be underpowered to detect a difference of 10% in the index. In other words, replacing 1/5 of the dietary oil may not affect the cardiovascular risk factors theoretically.

## Conclusions

In conclusion in our study, there are no significant differences in the cardiovascular risk factors after dietary intervention with palm olein. Considering the composition of dietary oil, we can conclude that palm olein with high ratio of saturated fatty acids in dietary oil may not affect the lipid profiles, the insulin resistance, and the function of liver and kidney, especially the oxidative stress level. However, there are still several limitations in this study. Firstly, the intervention time is 16 weeks, which is not long enough to observe the long-term influence of palm olein. And convenience sampling has been used in this study; therefore the results may not be suitable for all adults. Secondly, the sample size may not be powerful to observe the difference between three groups and we will enlarge the sample size to further investigate the effect of palm olein in the follow-up study. Furthermore, it is still contradictory on dietary saturated fatty acids and needs further investigation.

## Supplementary Material

Effects of dietary palm olein on the cardiovascular risk factors in healthy young adults
