# Supplementary material for: Effects of dietary palm olein on the cardiovascular risk factors in healthy young adults
Source: Food Nutr Res. 2018 Jul 16;62:10.29219/fnr.v62.1353. doi: 10.29219/fnr.v62.1353 (PMC6052506; doi:10.29219/fnr.v62.1353)
Supplement: Effects of dietary palm olein on the cardiovascular risk factors in healthy young adults [file FNR-62-1353-s001.pdf]

Table S1 The macronutrient and energy composition of the tested meals

Name	Meals	Weight(g)	Energy (kj)	Energy (kcal)	Protein (g)	Fat (g)	Carbohydrate (g)
**Monday**							
Steamed bun with meat stuffing	Breakfast	70	905.45	216.41	7.07	13.34	17.29
Egg	Breakfast	65	344.34	82.30	7.61	5.03	1.60
Stirabout	Breakfast	291	552.90	132.15	4.07	2.04	24.44
Milk	Breakfast	200	452.00	108.03	6.00	6.40	6.80
Red bean pastry	Lunch	125	2040.75	487.75	9.89	19.19	69.91
Steamed rice	Lunch	128	622.08	148.68	3.33	0.38	33.15
Stewed Pork Bone	Lunch	78	542.46	129.65	8.62	8.36	4.91
Fried cabbage	Lunch	183	303.60	72.56	2.68	4.64	5.71
Steamed rice	Dinner	130	631.80	151.00	3.38	0.39	33.67
Stewed Pork Bone	Dinner	78	542.46	129.65	8.62	8.36	4.91
Rape fried mushrooms	Dinner	291	583.51	139.46	5.20	11.13	9.08
Total			7521.35	1797.65	66.47	79.26	211.49
**Tuesday**							
Cantonese sponge cake	Breakfast	166	2721.95	650.56	11.67	20.88	104.81
Egg	Breakfast	54	286.07	68.37	6.32	4.18	1.33
Rice porridge	Breakfast	349	663.10	158.48	4.89	2.44	29.32
Milk	Breakfast	200	452.00	108.03	6.00	6.40	6.80
Steamed twisted roll	Lunch	73	653.35	156.15	4.67	0.73	33.29
Steamed rice	Lunch	130	631.80	151.00	3.38	0.39	33.67

Fried pork with scrambled eggs and fungus	Lunch	215	1241.53	296.73	11.79	24.86	6.85
Rape fried mushrooms	Lunch	217	435.13	104.00	3.88	6.77	8.30
Steamed rice	Dinner	130	631.80	151.00	3.38	0.39	33.67
Fried chicken with green pepper	Dinner	212	663.66	158.62	12.71	9.04	7.20
Fried bean sprout with leek.	Dinner	186	469.02	112.10	8.02	5.85	8.24
Total			8849.41	2115.06	505.51	120.82	28.88
**Wednesday**							
Chinese style pork floss bread	Breakfast	97	1828.70	437.07	9.66	19.70	56.04
Egg	Breakfast	65	344.34	82.30	7.61	5.03	1.60
Mung bean porridge	Breakfast	537	1020.30	243.86	7.52	3.76	45.11
Steamed twisted roll	Lunch	73	653.35	156.15	4.67	0.73	33.29
Steamed rice	Lunch	130	631.80	151.00	3.38	0.39	33.67
Scrambled eggs with lettuce	Lunch	244	561.20	134.13	6.00	9.60	6.53
Fried pork with scrambled eggs and fungus	Lunch	215	1241.53	296.73	11.80	24.86	6.85
Steamed rice	Dinner	130	631.80	151.00	3.38	0.39	33.67
Braised beaf with potato	Dinner	300	1310.29	313.17	24.07	11.83	28.49
Small shrimps fried spinach	Dinner	205	420.77	100.57	5.25	5.59	9.00
Total			8644.09	2065.99	83.33	81.89	254.24
**Thursday**							
Ujube cake	Breakfast	78	1379.69	329.75	5.44	21.20	29.49
Steamed bun with meat stuffing	Breakfast	70	905.45	216.41	7.07	13.34	17.29
Milk	Breakfast	200	452.00	108.03	6.00	6.40	6.80

Steamed rice	Lunch	130	631.80	151.00	3.38	0.39	33.67
Steamed chicken leg with black bean sauce.	Lunch	215	1208.97	288.95	23.44	21.70	0
Garlic lettuce	Lunch	270	455.50	108.87	3.41	8.64	5.24
Steamed rice	Dinner	131	636.66	152.17	3.41	0.39	33.93
Braised beaf with potato	Dinner	300	1310.29	313.17	24.07	11.83	28.49
Steamed egg custard	Dinner	239	1327.42	317.26	27.75	20.26	5.84
Total			8307.78	1985.61	103.97	104.14	160.75
**Friday**							
Walnut cake	Breakfast	70	1487.61	355.55	4.31	21.68	36.18
Egg	Breakfast	65	344.34	82.30	7.61	5.03	1.60
Steamed bun with meat stuffing	Breakfast	70	905.45	216.41	7.07	13.34	17.29
Milk	Breakfast	200	452.00	108.03	6.00	6.40	6.80
Tomato fried cauliflower	Lunch	248	400.84	95.80	1.98	6.24	9.56
Steamed fish fillets	Lunch	153	494.79	118.26	14.52	6.70	0
Steamed rice	Lunch	142	690.12	164.94	3.69	0.43	36.78
Steamed rice	Dinner	142	690.12	164.94	3.69	0.43	36.78
Diced chicken in bean sauce	Dinner	194	891.41	213.05	15.08	14.85	5.01
Fried tofu with Minced pork	Dinner	258	1341.88	320.72	21.70	21.65	10.23
Total			7698.56	1840.00	85.66	96.73	160.23
